# Association of Simulated COVID-19 Vaccination and Nonpharmaceutical Interventions With Infections, Hospitalizations, and Mortality

**DOI:** 10.1001/jamanetworkopen.2021.10782

**Published:** 2021-06-01

**Authors:** Mehul D. Patel, Erik Rosenstrom, Julie S. Ivy, Maria E. Mayorga, Pinar Keskinocak, Ross M. Boyce, Kristen Hassmiller Lich, Raymond L. Smith, Karl T. Johnson, Paul L. Delamater, Julie L. Swann

**Affiliations:** 1 Department of Emergency Medicine, School of Medicine, University of North Carolina at Chapel Hill; 2 Department of Industrial and Systems Engineering, North Carolina State University, Raleigh; 3 Department of Industrial and Systems Engineering, Georgia Institute of Technology, Atlanta; 4 Division of Infectious Diseases, School of Medicine, University of North Carolina at Chapel Hill; 5 Department of Health Policy and Management, Gillings School of Global Public Health, University of North Carolina at Chapel Hill; 6 Department of Engineering, College of Engineering and Technology, East Carolina University, Greenville, North Carolina; 7 Department of Geography, College of Arts and Sciences, University of North Carolina at Chapel Hill

## Abstract

**Importance:**

Vaccination against SARS-CoV-2 has the potential to significantly reduce transmission and COVID-19 morbidity and mortality. The relative importance of vaccination strategies and nonpharmaceutical interventions (NPIs) is not well understood.

**Objective:**

To assess the association of simulated COVID-19 vaccine efficacy and coverage scenarios with and without NPIs with infections, hospitalizations, and deaths.

**Design, Setting, and Participants:**

An established agent-based decision analytical model was used to simulate COVID-19 transmission and progression from March 24, 2020, to September 23, 2021. The model simulated COVID-19 spread in North Carolina, a US state of 10.5 million people. A network of 1 017 720 agents was constructed from US Census data to represent the statewide population.

**Exposures:**

Scenarios of vaccine efficacy (50% and 90%), vaccine coverage (25%, 50%, and 75% at the end of a 6-month distribution period), and NPIs (reduced mobility, school closings, and use of face masks) maintained and removed during vaccine distribution.

**Main Outcomes and Measures:**

Risks of infection from the start of vaccine distribution and risk differences comparing scenarios. Outcome means and SDs were calculated across replications.

**Results:**

In the worst-case vaccination scenario (50% efficacy, 25% coverage), a mean (SD) of 2 231 134 (117 867) new infections occurred after vaccination began with NPIs removed, and a mean (SD) of 799 949 (60 279) new infections occurred with NPIs maintained during 11 months. In contrast, in the best-case scenario (90% efficacy, 75% coverage), a mean (SD) of 527 409 (40 637) new infections occurred with NPIs removed and a mean (SD) of 450 575 (32 716) new infections occurred with NPIs maintained. With NPIs removed, lower efficacy (50%) and higher coverage (75%) reduced infection risk by a greater magnitude than higher efficacy (90%) and lower coverage (25%) compared with the worst-case scenario (mean [SD] absolute risk reduction, 13% [1%] and 8% [1%], respectively).

**Conclusions and Relevance:**

Simulation outcomes suggest that removing NPIs while vaccines are distributed may result in substantial increases in infections, hospitalizations, and deaths. Furthermore, as NPIs are removed, higher vaccination coverage with less efficacious vaccines can contribute to a larger reduction in risk of SARS-CoV-2 infection compared with more efficacious vaccines at lower coverage. These findings highlight the need for well-resourced and coordinated efforts to achieve high vaccine coverage and continued adherence to NPIs before many prepandemic activities can be resumed.

## Key Points

**Question:**

What is the association of COVID-19 vaccine efficacy and coverage scenarios with and without nonpharmaceutical interventions (NPIs) with SARS-CoV-2 infections, hospitalizations, and deaths?

**Findings:**

A decision analytical model of North Carolina found that removing NPIs while vaccines were distributed resulted in substantial increases in infections, hospitalizations, and deaths. Furthermore, as NPIs were removed, higher vaccination coverage with less efficacious vaccines contributed to a larger reduction in risk of infection compared with more efficacious vaccines at lower coverage.

**Meaning:**

These findings highlight the need for high COVID-19 vaccine coverage and continued adherence to NPIs before safely resuming many prepandemic activities.

## Teaser

This decision analytical study assesses the association of simulated COVID-19 vaccine efficacy and coverage scenarios with and without nonpharmaceutical interventions with infections, hospitalizations, and deaths.

## Introduction

SARS-CoV-2 vaccines will play a major role in achieving sufficient population immunity to end the COVID-19 pandemic.^1^ In May 2020, the US Department of Health and Human Services launched Operation Warp Speed, a public-private partnership with the goal of administering 300 million doses of COVID-19 vaccine by January 2021.^2^ Furthermore, with an estimated 16 billion doses required to meet global demand, mass vaccination campaigns across the world will be unprecedented.^3^ In December 2020, the Moderna^4^ and Pfizer-BioNTech^5^ messenger RNA vaccines were authorized for emergency use, with more expected to follow in 2021.^6,7^

Although the primary end point of COVID-19 vaccine trials is efficacy to prevent severe disease and death, these vaccines are expected to reduce transmission and contribute to population immunity required to end the pandemic.^8^ Recent mathematical modeling suggests that prioritizing vaccine distribution and uptake would maximize the benefit of a highly efficacious vaccine.^8,9,10^ Bartsch et al^9^ estimated that at least 75% of the US population would need to be vaccinated with an efficacy of 70% to reduce the epidemic peak by more than 99% without other interventions. Considering the complexities of large-scale vaccine production, distribution, and uptake, achieving high coverage will be challenging.^9^ Therefore, critical questions remain regarding the need to continue nonpharmaceutical interventions (NPIs), such as physical distancing and use of face masks, as the public is vaccinated over time.^8,11^ Models of SARS-CoV-2 transmission that capture complex and heterogeneous interactions between individuals are needed to understand the dynamics between COVID-19 vaccination strategies and other behavioral interventions.^12^

Using an integrated compartmental disease transmission model and agent-based simulation,^13,14,15^ we estimated the effect of hypothetical scenarios of vaccine efficacy and population coverage on SARS-CoV-2 infections and COVID-19–related hospitalizations and deaths. Simulating vaccine distribution in North Carolina, we compared the outcomes of varying efficacy and coverage with NPIs maintained and removed concurrently with vaccine distribution. To explore meaningful differences between subpopulations, we stratified the results by race/ethnicity and urban, suburban, and rural communities. These objectives were informed by state and local public health decision-makers involved in the COVID-19 response.

## Methods

### Study Design

In this decision analytical study, we used an agent-based stochastic network with a susceptible-exposed-infectious-recovered (SEIR) framework for the transmission and progression of SARS-CoV-2 infections.^13,14,15^ Agent-based models with an SEIR framework have been widely used and accepted for modeling disease spread while considering population behavior.^16,17,18^ In this SEIR model, an individual can be in one of the following states at any given time: susceptible, exposed, presymptomatic infectious, asymptomatic infectious, symptomatic infectious, severe disease requiring hospitalization, and recovered. Individuals who recover from infection permanently remain recovered and do not return to a susceptible state. Using the methods of Keskinocak et al,^14^ we simulated heterogeneous mixing and interactions among agents where daily transmission occurs within households, workplaces, schools, and communities (census tracts). The network of agents was constructed using US Census tract-level data on household size (number of adults and children), age groups (0-4, 5-9, 10-19, 20-64, and ≥65 years), and race/ethnicity. Model parameters include the basic reproductive number (R_0_ = 2.4),^19,20,21^ asymptomatic and symptomatic transmission coefficients,^19,22^ and age-specific hospitalization and mortality rates.^23^ Diabetes was included as a risk factor for hospitalization conditional on individual age and race as derived from statewide prevalence data.^24^ Model details and parameter values are provided in eMethods, eFigures 1 to 3, and eTable 1 in the Supplement. This study was determined exempt from institutional review board oversight and the requirement of informed consent by the University of North Carolina Office of Human Research Ethics. We followed the Consolidated Health Economic Evaluation Reporting Standards (CHEERS) reporting guideline.

### Study Population

The state of North Carolina was selected as a representative region, with a population of more than 10 million and diversity across age, race/ethnicity, and socioeconomic groups. According to US Census estimates for North Carolina, 18% of residents are 65 years or older, 20% are Black or African American individuals, 7% are Hispanic or Latino individuals, and 19% live in households earning less than the poverty threshold set by the US Census Bureau.^25^ Of the 100 North Carolina counties, 80 are rural, with a mean population density of 250 people or fewer per square mile.^26^ As of December 21, 2020, North Carolina was ranked 38th in the country for deaths per capita, with approximately 60 total deaths per 100 000 residents.^27^ A statewide stay-at-home order was in place from March 24 through May 8, 2020, and face masks have been required in indoor public settings since June 24, 2020. As of August 24, 2020, kindergarten through grade 12 schools have been open for both reduced occupancy in-person and remote learning, with local school districts determining the operating model, most of which are hybrid.^28^

### Agent-Based Simulation

The North Carolina population of 10 490 000, which consists of 2195 census tracts, was modeled with 1 017 720 agents. The simulation was seeded (day 1) with cumulative infections as of March 24, 2020,^27^ with the laboratory-confirmed cases multiplied by a factor of 10 to account for limited testing and underreporting^29^ to approximate the true number of infections. This number was then scaled to the number of agents in the simulation. The simulation spanned 18 months (548 days; from March 24, 2020, to September 23, 2021) to evaluate the association of vaccination scenarios with key outcomes for the current pandemic. Model outcomes were scaled back to the total North Carolina population. The model was calibrated and validated using the number of laboratory-confirmed cases and reported deaths as of November 1, 2020 (eMethods and eTable 2 in the Supplement). County-specific cases were randomly distributed to households across the county’s tracts proportionally to the tract population. For each simulated scenario, 45 replications were performed. A different seed population (ie, agent demographic characteristics and who they interact with and where) was selected for each replication and accounts for variation across replications. For subgroup analyses, simulation results were stratified by race/ethnicity (non-Hispanic White, non-Hispanic Black or African American, and Hispanic). Results were not reported for other races, such as Asian, Native American, and multiracial, because of small denominators. We stratified by urban, suburban, and rural tracts defined by rural-urban commuting area codes (1-2, 3-5, and 6-10, respectively).

### COVID-19 Vaccination

In simulations, vaccines were distributed uniformly to adults (20 years and older) during a 6-month period. We simulated scenarios in which vaccine distribution started when 10% of the state had been infected (day 213). Three vaccine coverage scenarios achieved 25%, 50%, and 75% of adults vaccinated by the end of the 6-month distribution period. Vaccine distribution ramped up with 50% of the final coverage distributed in the first 4 months and 50% distributed in the last 2 months. Vaccine efficacy was modeled as the probability that a vaccinated, susceptible agent directly transitions to the recovered state. In this model, as with individuals who recover from infection, vaccinated individuals who transition permanently remain recovered. We assumed the vaccine conferred immunity immediately. Vaccine efficacies tested were 90%, based on preliminary estimates of vaccine efficacy from phase 3 trials, and 50%, based on the minimum efficacy threshold established by the US Food and Drug Administration. We simulated the 2 efficacy levels and 3 coverage levels, resulting in 6 vaccination scenarios (A-F), the best case being 90% efficacy with 75% coverage (A), and the worst case being 50% efficacy with 25% coverage (F). In addition, we simulated a no-vaccine scenario (G) to provide context.

### NPIs

Limited mobility (ie, physical distancing) was estimated with SafeGraph mobility data^30^ aggregated by census tract rural-urban commuting area code and income quartile. Data reflected the stay-at-home order in place from March 24 to May 8, 2020, for nonessential workers as well as reductions in distancing over time. We also modeled voluntary isolation and quarantine where an agent with symptomatic infection isolated at home with all members of the household until the agent recovered. Based on mobility data and computational experiments, this scenario occurred with 60% probability initially and then decreased by 15 percentage points each week until reaching 15%, where it remained for the duration of the simulation.

Use of face masks among agents began at 0 and increased at a constant rate to 70% when face masks were mandated statewide in June. Masks were assumed to reduce exposure to infection and infectivity among susceptible agents by 50% each.^31,32^ Because schools closed in March and moved to remote instruction, there were no kindergarten through grade 12 school interactions until August 24, when schools were allowed to open under a hybrid policy that limited the total number of students to ensure distancing. After August 24, agents were split into 2 equal groups, with one group attending school on even dates and the other on odd dates.

**Figure 1.**
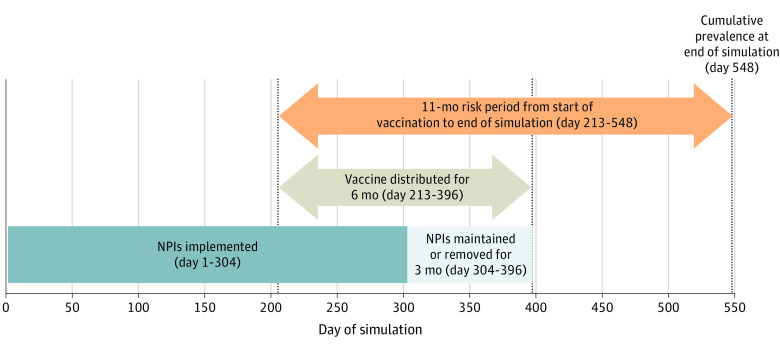
Description of Vaccination and Nonpharmaceutical Interventions (NPIs) During the 18-Month Simulation The agent-based model simulated an 18-month period, with vaccines distributed during 6 months and NPIs implemented. Midway through vaccine distribution, NPIs were either maintained or removed. Outcomes were computed across the entire simulation and from the start of vaccination to the end of the simulation.

We simulated vaccination scenarios (A-F) with these NPIs maintained (A1-F1) and removed (A0-F0). When NPIs were maintained, they remained at prevaccination levels throughout the simulation. When NPIs were removed, workplace and community interactions, including schooling, returned to normal and agents no longer isolated or quarantined immediately at the midpoint of the 6-month vaccine distribution period. Use of face masks decreased at a rate proportional to the number of agents vaccinated until the end of vaccine distribution. Figure 1 shows the timing of interventions during the 18-month simulation.

#### Statistical Analysis

The outcomes estimated included daily total infections (symptomatic and asymptomatic), hospitalizations, and deaths. For each outcome, we computed totals during the simulation period and cumulative incidence on the final day of the simulation (Figure 1). To compare the effects of different vaccination scenarios with NPIs maintained (A1-F1) and NPIs removed (A0-F0), we computed risks of infection from the start of vaccine distribution and risk differences between scenarios, with the worst-case vaccination scenario with NPIs removed (F0) as the reference category. The mean and SD of each outcome measure were calculated across the 45 replications. The simulation model was implemented in C++ (version 11), and analyses were conducted in Python (version 3.7.7).

#### Sensitivity Analysis

To evaluate the robustness of results to parameter specification, we conducted a 2-way sensitivity analysis in which the transmission rate (β) and mask efficacy were varied. The original transmission rate of 1.12 was set to lower and upper bounds (1.06 and 1.19, respectively) per Li et al.^19^ The original mask efficacy of 50% was also varied to 70% (based on a recent evidence review^33^) and 30% (as a more conservative estimate of protection). Under these combinations of parameter values, simulations were run for the A1, F1, A0, and F0 scenarios, and the estimated risk differences were compared with those from the main analysis.

## Results

### Statewide Analysis

**Figure 2.**
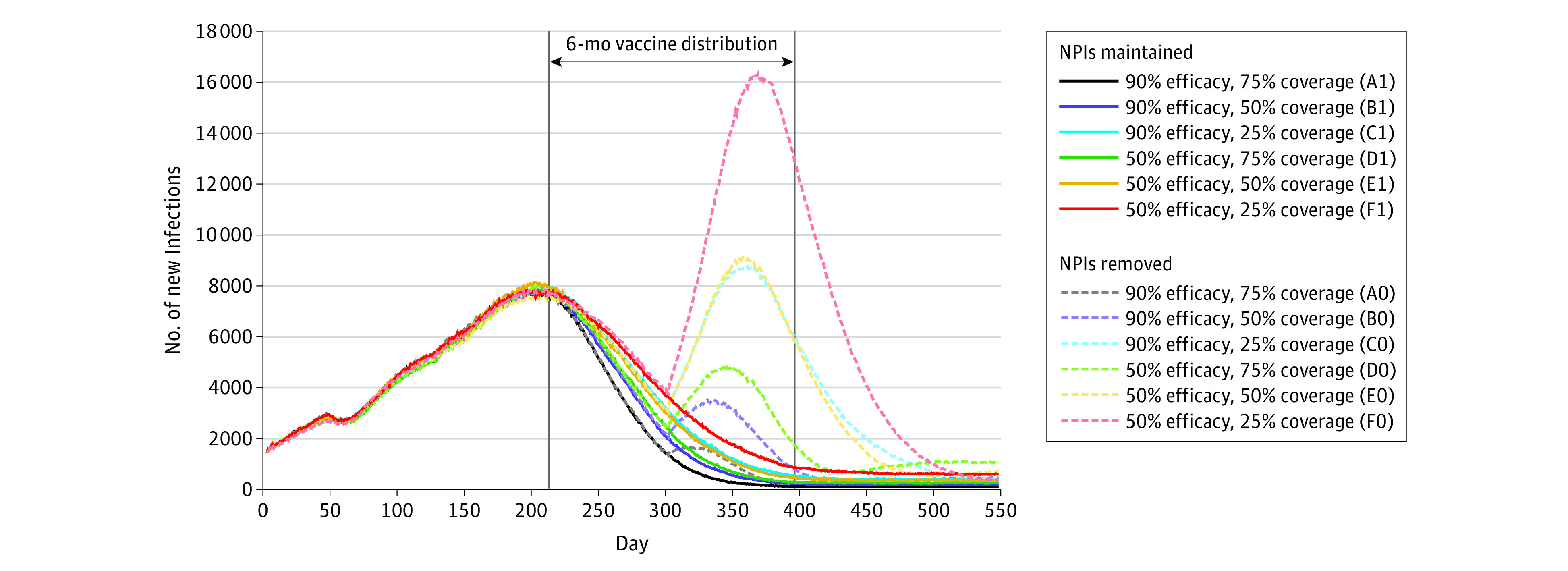
Daily New Infections by Vaccination and Nonpharmaceutical Intervention (NPI) Scenarios During the 18-Month Simulation Modeled new infections by day are shown across varying vaccine efficacy and coverage with NPIs maintained and removed.

In a simulation of 10 490 000 individuals, a 50% efficacious vaccine at 25% coverage with NPIs removed (F0) resulted in a mean (SD) infection rate of 30.8% (0.2%) after 18 months (Table 1). During the 11-month period from the onset of vaccination, a mean (SD) of 2 231 134 (117 867) new infections occurred in this worst-case scenario compared with a mean (SD) of 799 949 (60 279) infections with NPIs maintained. In contrast, the best-case 90% efficacious vaccine at 75% coverage with NPIs maintained (A1) resulted in a mean (SD) of 450 575 (32 716) new infections, for a 19% (SD, 1%) absolute risk reduction compared with a mean (SD) of 527 409 (40 637) new infections with NPIs removed. When NPIs were removed, mean (SD) risk reductions ranged from 9% (1%) for E0 to 18% (1%) for A0 with increasing vaccine efficacy and coverage. Scenario D0 (50% efficacy with 75% coverage) had a greater risk reduction than scenario C0 (90% efficacy with 25% coverage) at a mean (SD) of 13% (1%) and 8% (1%), respectively, suggesting vaccine coverage had a greater association with reducing infections than efficacy. Figure 2 shows more pronounced differences in daily new infections with and without NPIs with lower vaccine efficacy and coverage. For example, scenarios with NPIs removed showed second infection peaks, whereas those with NPIs maintained did not (eg, under the F0 scenario, an estimated 16 335 new infections occurred on day 368).

**Table 1.** Risk of SARS-CoV-2 Infection From Start of Vaccine Distribution by Vaccination and NPI Scenarios

Scenario	Vaccine characteristic		NPI condition	Mean (SD)			
				New infections, No.^a^	Risk^a^	Risk difference^b^	Cumulative infection rate, %^c^
	Efficacy, %	Coverage, %					
A1	90	75	Maintained	450 575 (32 716)	0.047 (0.003)	−0.19 (0.01)	13.8 (0.2)
B1		50		542 261 (38 087)	0.057 (0.004)	−0.18 (0.01)	14.8 (0.2)
C1		25		698 192 (50 601)	0.074 (0.005)	−0.16 (0.01)	16.3 (0.2)
D1	50	75		586 246 (48 548)	0.062 (0.005)	−0.17 (0.01)	15.1 (0.2)
E1		50		672 970 (46 489)	0.071 (0.005)	−0.16 (0.01)	16.2 (0.2)
F1		25		799 949 (60 279)	0.084 (0.006)	−0.15 (0.01)	17.3 (0.2)
G1	No vaccine	No vaccine		1 100 664 (61 891)	0.116 (0.006)	−0.12 (0.01)	19.9 (0.2)
A0	90	75	Removed	527 409 (40 637)	0.056 (0.004)	−0.18 (0.01)	14.7 (0.2)
B0		50		748 572 (57 950)	0.079 (0.006)	−0.16 (0.01)	16.6 (0.2)
C0		25		1 441 841 (95 010)	0.152 (0.009)	−0.08 (0.01)	23.3 (0.2)
D0	50	75		957 149 (89 784)	0.101 (0.009)	−0.13 (0.01)	18.6 (0.2)
E0		50		1 407 563 (103 155)	0.148 (0.010)	−0.09 (0.01)	22.9 (0.2)
F0, reference		25		2 231 134 (117 867)	0.235 (0.011)	0.00 (0.02)	30.8 (0.2)
G0	No vaccine	No vaccine		3 879 088 (125 243)	0.409 (0.009)	0.17 (0.01)	46.5 (0.2)

Abbreviation: NPI, nonpharmaceutical intervention.

^a^ From start of vaccine distribution (day 213, 10% cumulative infection prevalence among a population of 10.49 million) to end of simulation (11.0 months).

^b^ Indicates absolute difference in risk compared with the worst-case vaccination scenario with NPIs removed (F0).

^c^ Through end of simulation (day 548).

All vaccination scenarios with NPIs had lower risks than without NPI counterparts; these differences tended to increase with lower vaccine efficacy and coverage. Maintaining NPIs with the worst-case vaccination scenario (F1) reduced infections by a mean (SD) of 15% (1%) compared with this scenario without NPIs (F0). In contrast, the mean (SD) risk reductions for scenarios A1 and A0 were similar at 19% (1%) vs 18% (1%), suggesting that NPIs had a smaller association with outcomes in best-case vaccination. Similar patterns for the joint association of vaccination and NPIs were observed for mortality risks and hospitalizations. A mean (SD) of 15 166 (589) deaths, accounting for 0.1% of the population, resulted from worst-case vaccination without NPIs (F0), whereas best-case vaccination with NPIs (A1) resulted in a mean (SD) of 6789 (450) total deaths (Table 2).

**Table 2.** COVID-19 Mortality and Hospitalizations by Vaccination and NPI Scenarios

Scenario	Vaccine characteristic		NPI condition	Mean (SD)					Cumulative hospitalization per 100 000 population^c^
				New deaths, No.^a^	Mortality risk (per 100 000)	Mortality risk difference^b^	Total deaths, No.^c^	Total hospitalizations, No.^c^	
	Efficacy, %	Coverage, %							
A1	90	75	Maintained	2777 (260)	26.5 (2.5)	−79.8 (7.0)	6789 (450)	52 167 (1086)	497
B1		50		3227 (244)	30.7 (2.3)	−75.6 (6.9)	7265 (405)	55 924 (1109)	533
C1		25		3943 (301)	37.6 (2.9)	−68.7 (7.1)	7981 (430)	61 593 (1138)	587
D1	50	75		3364 (320)	32.1 (3.1)	−74.2 (7.2)	7374 (415)	56 852 (1114)	542
E1		50		3832 (310)	36.5 (3.0)	−69.8 (7.1)	7935 (406)	61 111 (1134)	583
F1		25		4465 (369)	42.6 (3.5)	−63.7 (7.4)	8508 (442)	65 632 (1160)	626
G1	No vaccine	No vaccine		5760 (405)	54.9 (3.9)	−51.4 (7.6)	9721 (425)	75 549 (1214)	720
A0	90	75	Removed	3120 (287)	29.7 (2.7)	−76.6 (7.0)	7230 (463)	55 160 (1097)	526
B0		50		4207 (324)	40.1 (3.1)	−66.2 (7.2)	8153 (388)	61 855 (1128)	590
C0		25		7510 (527)	71.6 (5.0)	−34.7 (8.2)	11 511 (464)	87 656 (1230)	836
D0	50	75		5109 (508)	48.7 (4.8)	−57.6 (8.1)	9081 (381)	69 184 (1154)	660
E0		50		7282 (491)	69.5 (4.7)	−36.8 (8.0)	11 258 (481)	85 290 (1221)	813
F0, reference		25		11 152 (683)	106.3 (6.5)	0.0 (9.2)	15 166 (589)	116 060 (1318)	1106
G0	No vaccine	No vaccine		18 662 (570)	177.9 (5.4)	71.6 (9.2)	22 659 (446)	178 409 (1473)	1701

Abbreviation: NPI, nonpharmaceutical intervention.

^a^ From start of vaccine distribution (day 213, 10% cumulative infection prevalence among a population of 10.49 million) to end of simulation (11.0 months).

^b^ Indicates absolute difference in risk (per 100 000 population) compared with the worst-case vaccination scenario (F0) with NPIs removed.

^c^ Through end of simulation (day 548).

### Subgroup Analyses

Cumulative incidences of infections, hospitalizations, and deaths by scenario varied across racial/ethnic groups (eTable 3 in the Supplement). White individuals had the lowest incidence of infections, whereas Hispanic individuals had the lowest hospitalization and mortality rates. Black individuals tended to have the highest incidence of infections, hospitalizations, and mortality. The mean (SD) reduction with best-case vaccination and maintaining NPIs (scenarios A1 vs F0) was greater among White and Black individuals (−82 [6] and −82 [10], respectively, per 100 000) compared with Hispanic individuals (−72 [14] per 100 000) (Table 3). This trend in mortality reduction between racial/ethnic groups was generally consistent for each vaccination-NPI scenario according to difference-in-differences analyses.

**Table 3.** COVID-19 Mortality by Vaccination and NPI Scenarios Across Racial/Ethnic Groups and Urban-Rural Tracts

Scenario	Vaccine characteristic		NPI condition	Mean (SD)											
				Racial/ethnic group						Rural-urban classification^a^					
				White		Black		Hispanic		Urban		Suburban		Rural	
	Efficacy, %	Coverage, %		Cumulative mortality per 100 000 population^b^	Mortality difference^c^	Cumulative mortality per 100 000 population	Mortality difference	Cumulative mortality per 100 000 population	Mortality difference	Cumulative mortality per 100 000 population	Mortality difference	Cumulative mortality per 100 000 population	Mortality difference	Cumulative mortality per 100 000 population	Mortality difference
A1	90	75	Maintained	64 (3)	−82 (6)	73 (6)	−82 (10)	60 (8)	−72 (14)	62 (1)	−76 (1)	75 (2)	−82 (4)	72 (3)	−98 (5)
B1		50		68 (3)	−78 (6)	79 (6)	−76 (10)	64 (8)	−69 (14)	66 (1)	−73 (1)	78 (2)	−79 (4)	85 (3)	−85 (5)
C1		25		75 (4)	−71 (6)	86 (6)	−68 (10)	69 (9)	−64 (15)	72 (1)	−66 (1)	89 (3)	−68 (4)	88 (3)	−82 (5)
D1	50	75		69 (3)	−76 (6)	80 (6)	−75 (10)	66 (9)	−66 (15)	67 (1)	−71 (1)	79 (2)	−77 (4)	85 (3)	−86 (5)
E1		50		75 (4)	−71 (6)	85 (6)	−70 (10)	70 (9)	−63 (15)	72 (1)	−66 (1)	88 (3)	−69 (4)	88 (3)	−83 (5)
F1		25		81 (4)	−65 (6)	90 (6)	−64 (10)	72 (9)	−60 (15)	77 (1)	−62 (1)	94 (3)	−63 (4)	98 (3)	−73 (5)
G1	No vaccine	No vaccine		92 (4)	−53 (6)	102 (7)	−53 (11)	86 (10)	−47 (16)	88 (1)	−51 (1)	107 (3)	−50 (4)	112 (4)	−59 (6)
A0	90	75	Removed	68 (3)	−78 (6)	78 (6)	−77 (10)	65 (9)	−67 (15)	65 (1)	−74 (1)	82 (3)	−75 (4)	85 (3)	−86 (5)
B0		50		77 (4)	−69 (6)	87 (7)	−68 (11)	74 (9)	−58 (15)	74 (1)	−65 (1)	87 (3)	−70 (4)	91 (3)	−80 (5)
C0		25		110 (4)	−35 (6)	119 (7)	−36 (11)	99 (11)	−33 (16)	106 (1)	−32 (1)	123 (3)	−34 (4)	127 (4)	−43 (6)
D0	50	75		86 (4)	−60 (6)	95 (7)	−59 (11)	81 (9)	−52 (15)	83 (1)	−55 (1)	83 (3)	−73 (4)	99 (3)	−71 (5)
E0		50		108 (4)	−37 (6)	115 (7)	−39 (11)	96 (10)	−37 (16)	102 (1)	−36 (1)	116 (3)	−41 (4)	121 (4)	−49 (6)
F0, reference		25		146 (5)	0 (7)	155 (8)	0 (11)	132 (12)	0 (17)	138 (1)	0 (1)	157 (3)	0 (4)	171 (4)	0 (6)
G0	No vaccine	No vaccine		220 (6)	74 (8)	226 (10)	71 (13)	194 (15)	61 (19)	206 (2)	67 (2)	243 (4)	86 (5)	260 (5)	90 (6)

Abbreviation: NPI, nonpharmaceutical intervention.

^a^ Census tract rural-urban commuting area code, with 1 to 2 indicating urban; 3 to 5, suburban; and 6 to 10, rural.

^b^ Through end of simulation (day 548).

^c^ Absolute difference in cumulative mortality (per 100 000 population) compared with the worst-case vaccination scenario (F0) with NPIs removed.

Rural tracts had a higher incidence of infections, hospitalizations, and deaths compared with urban tracts in all scenarios (eTable 4 in the Supplement). Although urban and suburban tracts had comparable cumulative infections, suburban tracts experienced more hospitalizations and higher mortality. The mean (SD) reduction with best-case vaccination and maintaining NPIs (scenarios A1 vs F0) increased across the urban-rural continuum (−76 [1] to −98 [5] per 100 000) (Table 3). Similar to race/ethnicity subgroup analyses, this general trend between tracts was consistent for each vaccination-NPI scenario.

### Sensitivity Analysis

Varying the parameter values for transmission rate and mask efficacy resulted in differences in infections risks while vaccines were distributed (eTable 5 in the Supplement). However, patterns in risk reductions were comparable with the main analysis and consistent with our results on the consequences of higher vaccine efficacy and coverage and maintaining NPIs during vaccine distribution.

## Discussion

Our study suggests that, for a population of 10.5 million, approximately 1.8 million infections and 8000 deaths could be prevented during 11 months with more efficacious COVID-19 vaccines, higher vaccination coverage, and maintaining NPIs, such as distancing and use of face masks. Moreover, our findings highlight the importance of continued adherence to NPIs while the population is vaccinated, particularly under scenarios of lower vaccine efficacy and coverage. Maintaining NPIs throughout the 6-month vaccine distribution period appeared to reduce infections to levels seen at the beginning of the pandemic. In contrast, under scenarios with low vaccine efficacy and coverage, premature removal of NPIs could result in a resurgence of infections with a magnitude exceeding that before vaccine distribution.

While initial vaccine trials have demonstrated 95% efficacy to reduce symptomatic infections,^34^ the ability of immunization to prevent asymptomatic infection and transmission is not well understood.^3,6,35^ Furthermore, protection may be limited in those who miss a booster dose or if a new strain of the virus with antibody resistance emerges.^36^ Therefore, a universal vaccine efficacy of 90% modeled in our study is likely to be overly optimistic. Similarly, vaccinating at least 75% of the adult population during 6 months, as modeled in our study, will be challenging given issues surrounding vaccine supply, distribution, and hesitancy.^37,38^ Thus, although the differences between the number of infections in the best-case vaccination scenario with and without NPIs were smaller than in other scenarios, achieving 90% efficacy and 75% coverage may not be realistic, emphasizing the need for continued NPI adherence.

Consistent with prior studies,^8,9^ our findings suggest that higher vaccine coverage (ie, 75% vs 25%) could lead to a comparatively greater reduction of infections than higher vaccine efficacy (ie, 90% vs 50%) when NPIs are relaxed. Currently, a strategy of reducing the number of doses of the messenger RNA vaccines to increase the vaccine supply and vaccinate more people is being debated.^39^ Although coverage is a function of vaccine supply, distribution, and uptake, it was only considered broadly in our study. Furthermore, the vaccine was distributed throughout the population uniformly rather than by priority groups. Comparing the scenarios with 90% efficacy and 25% coverage with those with 50% efficacy and 50% coverage, in which coverage is doubled, we observed minimal differences in risk of new infections and COVID-19–associated hospitalizations and deaths.

For SARS-CoV-2, the threshold for herd immunity is estimated at 58% for an R_0_ of 2.4, although depending on population characteristics and virus transmission dynamics,^40,41^ estimates can range from 43% to 90%.^42,43^ We found that sufficient population protection to slow new infections was achieved through vaccination during 6 months and NPIs, although removing NPIs during vaccination distribution was associated with additional hospitalizations and deaths. Our findings suggest that coordinated efforts are needed to maximize vaccine coverage and adherence to NPIs to reduce COVID-19 burden to a level that could safely allow a resumption of many economic, educational, and social activities.

We found disparities in COVID-19 mortality by race/ethnicity consistent with prior findings that Black/African American individuals are at higher risk of hospitalization and death.^44^ In our model, these disparities were partly explained by diabetes as a risk factor for hospitalization and were likely underestimated because we did not account for other high-risk conditions as well as the adverse effects of structural racism.^45,46^ We also found higher rates of COVID-19 hospitalizations and mortality in rural compared with urban communities. We did not account for the potential for hospitals or intensive care units to become overburdened, so deaths could be greater than estimated, particularly in rural areas. Assuming vaccine distribution is uniform, we found that vaccination and NPIs were associated with reduced infections, hospitalizations, and deaths across racial/ethnic groups and rurality, whereas no scenarios appeared to be substantially associated with reduced disparities in these outcomes. Although not examined herein, lower vaccine uptake among Black/African American and rural communities^38^ and the logistical challenges of vaccines requiring ultracold storage^47^ and multiple doses have the potential to further exacerbate disparities in COVID-19 mortality.

Unlike prior deterministic compartmental models of SARS-CoV-2 transmission and COVID-19 vaccination,^8,9,10,48,49,50^ our agent-based simulation model captured heterogeneous mixing between individuals and within households, schools, workplaces, and communities and allowed for joint consideration of various interventions. In this study, we did not intend to forecast COVID-19 burden for a specific population but to support public health officials by comparing the potential outcomes of vaccination and NPI scenarios to, for example, inform the public on the potential effects of vaccine uptake as well as continued adherence to existing mitigation strategies.^51^ Our agent-based modeling framework and others, such as JUNE,^12^ can be adapted to other locations using existing population data and publicly available data on infections and deaths. However, this approach may be less accurate for smaller regions, such as a single county, with relatively low numbers for model validation.

Our findings are conditional on our modeling assumptions and specification of vaccination and NPI scenarios. Notably, in our simulation, vaccination protected individuals from infection in an all-or-nothing manner (ie, a proportion of susceptible individuals transition to the recovered state), although partial protection could be more realistic.^48^ For this modeling, vaccination was simplified to confer protection immediately, although evidence shows there to be a delay.^4,5^ In interpreting the results, the start of vaccination can be considered the onset of protection due to vaccines instead of when the first dose was given. Our modeling also simplified vaccine distribution to a 6-month period for all coverages achieved. Moreover, distribution was uniform across the population and not prioritized by subgroups.^47,52^ We expect that phased allocation strategies that target high-risk groups while vaccine supplies are limited would initially lead to a greater reduction in hospitalizations and deaths but would have a lesser effect on total infections compared with our findings. Therefore, to control disease spread, a phased strategy would likely result in a greater need to maintain NPIs throughout vaccination. Finally, our model assumed immunity due to vaccination or recovery from natural infection was permanent. Recent studies found immunity to reinfection lasts at least 6 months,^53^ and neutralizing antibodies and other immune responses persist up to 8 months after infection.^54,55,56^ Given the available evidence and the period modeled, we feel this was a reasonable assumption. However, duration of protection after infection and vaccination remains unknown. If reinfections were more common than assumed, herd immunity would be more challenging to achieve, further emphasizing the need for increased vaccine coverage and maintaining NPIs. Future modeling will need to incorporate emerging evidence on long-term protection due to vaccination and recovery from natural infection.

### Limitations

In addition to the modeling assumptions discussed above, there are notable limitations to our study. The unique characteristics of North Carolina, including demographic characteristics, prevalence of diabetes, rurality, mobility, and adherence to NPIs, are associated with the transmission of SARS-CoV-2 and COVID-19 morbidity and mortality. General insights into the effect of vaccination and NPIs may apply to other states or regions, although exact numerical values will vary. The broad age category (20-64 years) limited age specificity of outcomes among adults. Demographic attributes (eg, age and race/ethnicity) within tracts were assigned independently, and interactions were not fully captured. Although our model projections followed major state-level counts and trends in cases, hospitalizations, and deaths, they did not capture all variability in reality, either over time or within subpopulations. Furthermore, although variability in outcomes across replications was calculated, our results do not reflect uncertainty in the model structure and parameter specification. However, sensitivity analysis suggests our main findings are robust to variability in important infection transmission and mitigation parameters.

## Conclusions

The results of this decision analytical study suggest that vaccinating the majority of the adult population and continued adherence to NPIs, such as distancing and use of face masks, will have the greatest effect on ending the current COVID-19 pandemic. These findings emphasize the need for resources and coordination to achieve high vaccine coverage and continued adherence to NPIs before safely resuming many prepandemic activities.

## Notes

Supplement. **eMethods.** Disease Model and Data Sources **eFigure 1.** Network of Agents Within Households, Schools, Workplaces, and Communities **eFigure 2.** Time-Varying Workplace Mobility Estimates by Census Tract Urban-Rural Classification and Median Household Income Quartile **eFigure 3.** SEIR Framework for Transition Between Model States **eTable 1.** Key Model Parameters **eTable 2.** Model Validation by Race and RUCA Subgroups **eTable 3.** Cumulative Incidence of Infections, Hospitalizations, and Deaths by Vaccination-NPI Scenario Across Racial/Ethnic Groups **eTable 4.** Cumulative Incidence of Infections, Hospitalizations, and Deaths by Vaccination-NPI Scenario Across Rural-Urban Classification **eTable 5.** Two-Way Sensitivity Analysis of the Risk of SARS-CoV-2 Infection From the Start of Vaccine Distribution by Vaccination-NPI Scenario Across Varying Transmission Rates and Mask Efficacies eReferences
