# Supplementary material for: Association of Simulated COVID-19 Vaccination and Nonpharmaceutical Interventions With Infections, Hospitalizations, and Mortality
Source: JAMA Netw Open. 2021 Jun 1;4(6):e2110782. doi: 10.1001/jamanetworkopen.2021.10782 (PMC8170542; doi:10.1001/jamanetworkopen.2021.10782)
Supplement: Supplement. — eMethods. Disease Model and Data Sources eFigure 1. Network of Agents Within Households, Schools, Workplaces, and Communities eFigure 2. Time-Varying Workplace Mobility Estimates by Census Tract Urban-Rural Classification and Median Household Income Quartile eFigure 3. SEIR Framework for Transition Between Model States eTable 1. Key Model Parameters eTable 2. Model Validation by Race and RUCA Subgroups eTable 3. Cumulative Incidence of Infections, Hospitalizations, and Deaths by Vaccination-NPI Scenario Across Racial/Ethnic Groups eTable 4. Cumulative Incidence of Infections, Hospitalizations, and Deaths by Vaccination-NPI Scenario Across Rural-Urban Classification eTable 5. Two-Way Sensitivity Analysis of the Risk of SARS-CoV-2 Infection From the Start of Vaccine Distribution by Vaccination-NPI Scenario Across Varying Transmission Rates and Mask Efficacies eReferences [file jamanetwopen-e2110782-s001.pdf]

Supplementary Online Content

Patel MD, Rosenstrom E, Ivy JS, et al. Association of simulated COVID-19 vaccination and nonpharmaceutical interventions with infections, hospitalizations, and mortality. *JAMA Netw Open*. 2021;4(6):e2110782. doi:10.1001/jamanetworkopen.2021.10782

eMethods. Disease Model and Data Sources

eFigure 1. Network of Agents Within Households, Schools, Workplaces, and Communities

eFigure 2. Time-Varying Workplace Mobility Estimates by Census Tract Urban-Rural Classification and Median Household Income Quartile

eFigure 3. SEIR Framework for Transition Between Model States

eTable 1. Key Model Parameters

eTable 2. Model Validation by Race and RUCA Subgroups

eTable 3. Cumulative Incidence of Infections, Hospitalizations, and Deaths by Vaccination-NPI Scenario Across Racial/Ethnic Groups

eTable 4. Cumulative Incidence of Infections, Hospitalizations, and Deaths by Vaccination-NPI Scenario Across Rural-Urban Classification

eTable 5. Two-Way Sensitivity Analysis of the Risk of SARS-CoV-2 Infection From the Start of Vaccine Distribution by Vaccination-NPI Scenario Across Varying Transmission Rates and Mask Efficacies

eReferences

This supplementary material has been provided by the authors to give readers additional information about their work.

© 2021 Patel MD et al. *JAMA Network Open*.

eMethods. Disease Model and Data Sources

In this document, we provide additional information on the model used to project the spread of COVID-19 infections and associated hospitalizations and deaths. The model was based on the structures of ones previously published for influenza [1-3] and COVID-19 [4-6] pandemics. The following documentation provides sufficient details for other researchers to reproduce our results or adapt this modeling to the populations or to address other questions.

Agent-Based Network

A network of agents allowed for interactions and transmission in households, workplace and school peer groups, and communities of census tracts (eFigure 1). The model used 1,017,720 agents to represent a statewide population of North Carolina (NC) of 10,490,000. Data values to generate individual agents were drawn from the US Census and were grouped into households. Census tract data was used to determine the size of the household (1 to 6), whether or not children were present [7,8], and the race/ethnicity of the household (with reporting for Black Only, White Only, Hispanic not White or Black, etc.) For the simulation, we assumed all individuals in the household are the same race/ethnicity and that it is independent of other factors. This last assumption was based on the census tract tables that were publicly available for recent years; it implies that our model is capturing less of the heterogeneity than exists in reality. Individual household members were assigned to age in five categories (age 0 to 4; age 5 to 9; age 10 to 19; age 20 to 64; age 65 and greater) [7], with the head of the household always 18 or older. As an indicator for high risk conditions, we used statewide prevalence of diabetes by race/ethnicity and adjusted for age [9]. The conditional probability of an agent having diabetes given their age and race can be calculated from the statewide prevalence values. While there are other conditions, diabetes affects many people and has a large impact on hospitalizations. During the day, all agents 5 to 19 interacted in peer groups ("schools"), and agents 19 to 64 interacted in peer groups ("work") according to commuting patterns. The latter was determined from workflow data indicating the people who live in a census tract and the percentage who work in each of the other census tracts [10]. When schools are closed (or hybrid), children stay at home (or stay home every other day). All agents interacted with household members at night.

For adult agents age 20 to 64, working from home or outside of the house was determined by time-varying mobility factors. Specifically, we used mobility data generated by SafeGraph, a data company that aggregates anonymized location data from numerous applications in order to provide insights about physical places, via the Placekey Community (Placekey.io) [11]. To enhance privacy, SafeGraph excludes census block group information if fewer than five devices visited an establishment in a month from a given census block group. SafeGraph tracks device location data and classifies each as working full time if that device spent greater than 6 hours at a location other than their home geohash-7 during the period of 8 am - 6 pm in local time. The data indicate the count of devices classified as working full-time. As in the publicly available Google mobility data [12], we computed the ratio of devices at home in comparison to the month of January to quantify the proportion of working-age adults staying home. Because of sparseness of data, we grouped points at the census tract level stratified by urban, rural, or suburban according to the Rural-Urban Commuting Area (RUCA) code and whether the tract was in the 1st, 2nd, 3rd, or 4th quartile of household median income for the state level. Beginning in month 6 we assume that the mobility factors stay the same. Based on the mobility data from SafeGraph and Google on mobility in contexts outside of work, we assume that the community mobility follows the same pattern as the workplace but to a lesser extent. For example, if the mobility is at 60% then the community factor is 88% of the original community value (specific calculation for new Community parameter: (1-((1-0.6)/3.5) * (community_parameter = 0.23) = 0.204). A reduction in the number of people in the community is modeled by a reduction in the community infectious hazard. In the workplace, the reduction of people follows the workplace mobility values. This decrease in workplace activity, reduces the workplace infectious hazard. Because of sparseness of data in rural areas, we grouped SafeGraph data at the census tract level stratified by urban, rural, or suburban according to the RUCA classification system and whether the tract was in the 1st, 2nd, 3rd, or 4th quartile of household median income for the state level (eFigure 2). To validate this approach, we compared to the Google mobility data available at the county level [12].

Disease Progression within an Individual

The underlying model of disease progression was assumed to be a variant of a Susceptible-Exposed-Infectious-Recovered (SEIR) model (eFigure 3). At a given point in time, each individual is in exactly one of the following states: susceptible (S), exposed (E), pre-symptomatic (IP), asymptomatic (IA), symptomatic (IS), hospitalized (IH), or recovered (R). The IH state represents disease severe enough to require hospitalization. In this model, individuals who recover from infection permanently remain recovered and do not return to a susceptible state. The list of input parameters and associated references is provided in eTable 1. Additional details are available in the supplement of Keskinocak, et al. [5].

Several parameters determine the length of time within each state in the SEIR including the mean and standard deviation on the time before an exposed patient becomes pre-symptomatic, the average length of time of the pre-symptomatic phase, the

© 2021 Patel MD et al. *JAMA Network Open*.

distribution of time within the symptomatic (S) stage, the distribution of the length of hospitalization, and the ratio of the duration of the symptomatic and asymptomatic states. Parameters related to the transmission between states include the probability of moving to symptomatic (from IP), the probability of hospitalization (from IS), and the probability of death (from IH); probabilities out of a state must sum to 100%. The probability of hospitalization or death is age-specific. The probability of hospitalization is also specific to whether someone has diabetes or not, matching both the relative risk and the overall hospitalization rate [13,14]. The overall Infection Fatality Rate (i.e., the probability that an exposed person will die) as quantified from the simulation of transitions is approximately 0.46%, which is consistent with estimates of IFR estimated by Hauser, et al. [15], which was also used for the CDC Pandemic Planning Scenarios.

The infectivity of the virus at the beginning of the outbreak without interventions is summarized by basic reproductive number R_0, and the transmission rate (denoted as β). The proportion of transmissions that occur at either the IP or IA stage is θ, and the proportion of infections generated by individuals who are never symptomatic is ω. In the absence of interventions, the proportion of transmission that occurs outside households is γ, and the proportion of transmission outside households that occur in the community is δ.

One of the important differences in comparison to the values used for influenza [1], is that the proportion of transmissions that can occur by people without symptoms is much higher. In comparison to the values used for the state of Georgia (4), δ is a little higher. For this paper we also take the import rate of cases to be lower (45 compared to 100) based on factors such as the airport size, commuting in or out of the state, etc.

Stochastic Simulation across Network

To seed infections in agents, the confirmed cases at the county level over time were collected from data hosted by The New York Times [29]. Initial infections (confirmed cumulative cases by March 24 times 10 [30]) were assigned to census tracts randomly according to the population within each tract in the county using the Huntington-Hill method.

The primary sources of randomness for the simulations include four types: (i) the structure of network, i.e., the random assignment of agents to households and peer groups (ii) the individual agents who are initially infected with the seed infections; (iii) whether an infected individual will transmit the virus to another person in the household, peer group, and/or community at a point in time; and (iv) the duration within a disease state. For each scenario, 45 replications of the simulation were run. The mean and standard deviation of each outcome was computed across the simulation replications. The number of replications was chosen so the mean values between scenarios have low variability until vaccine distribution begins.

Validation

The simulation output was scaled back to the total NC population, and the model was calibrated and validated on cumulative infections, hospitalizations, and deaths [31,32]. Deaths and hospitalizations are appropriate for direct validation; while not perfect, they are a reasonable estimate of true hospitalizations and deaths. However, it is well-recognized that the true cases are much greater than those that are lab-confirmed, and this value has changed over time (10-12 times [30] then later 8 times [33]). Using the Infection Fatality Rate (IFR) of 0.5% [15] and the average time from exposure to death (21 days), we computed the daily expected number of true cases that would have been needed to result in the recorded deaths. The average daily ratio of computed cases to lab-confirmed cases (or the "lab-multiplier") ranges from approximately 10 initially to 4 times (after 147 day). The estimates obtained in this way for one state and nationally are also consistent with CDC published accounts of antibody testing to identify the number of true cases [33]. This approach does not take into account the changing age-distribution of positive cases, which likely affected the timing of deaths. We compare our simulated results to reported deaths and hospitalizations and cases adjusted by the lab multiplier. For the baseline scenario without vaccine, our estimates are within 6% of the reported cumulative values.

While the model was validated using state-level data, we also assessed the validity of the model by comparing distributions of cases and deaths by race and RUCA subgroups (eTable 2). The race and RUCA distributions of cases from the model and NC data were generally comparable. Differences between cases may exist because the model estimated total infections (symptomatic and asymptomatic) with adjustments for under-testing whereas only lab-confirmed cases were publicly reported. Race distributions of deaths were also comparable. The model however estimated relatively fewer deaths in suburban tracts compared to reported data, which may be due to region-specific underreporting or incomplete residential information, perhaps earlier in the pandemic.

Simulated Interventions

The following interventions were analyzed as scenarios defined in the main text:

- <u>Vaccination:</u> Vaccine efficacy and the percentage of adults (20 year and older) receiving the vaccine ("coverage") determined the impact of vaccination.

© 2021 Patel MD et al. *JAMA Network Open*.

- Efficacy is defined as the percentage of those vaccinated who transition to recovered if they were exposed. Vaccinated individuals who transition to recovered permanently remain in that state. This implies that the vaccine can prevent both the symptomatic and asymptomatic states. For future research, we also developed a vaccine effect that shifts symptomatic infections to asymptomatic.
- Coverage is defined as the percent of the adult population who received the vaccine. Starting when the cumulative infection prevalence for the entire population was 10%, randomly selected adult agents (20 years or older) could be vaccinated regardless of disease state. The coverage parameter was reached over a 6-month distribution period. Initial vaccine supplies were assumed to be low. Based on expected supply, we assumed 18% of possible vaccinations occurred in months 0-2, 32% of possible vaccinations occurred in months 2-4, and 50% of vaccinations occurred in months 4-6. During each 2-month interval, the same amount of vaccine was distributed each week. Although coverage was modeled broadly here, it can be considered as a function of supply and uptake and modeled individually.

- <u>Non-Pharmaceutical Interventions (NPIs)</u>
    - Pre-Vaccination
        - Mobility was modeled using SafeGraph mobility data. Applying these data modeled the reduction in peer group activity for adults in the workplace and for all agents in the community setting. In NC, various orders were specified when the population should stay at home as much as possible including non-essential workers. We are capturing this through the realized mobility data over the time period studied. (See discussion above on agent mobility.) We assume that there are no additional orders that change the mobility after September.
        - Voluntary quarantine - if an agent had a family member that was symptomatic then the agent voluntarily quarantined until the agent has recovered. The probability of this behavior was higher during the first 50 days of the simulation. For the first 46 days the probability of voluntary quarantine was 0.3. At day 46 it was raised to 0.6. After day 46 it decreased by 0.15 per week until reaching 0.15. It remained at 0.15 for the duration of the simulation.
        - Schooling - Until August 24, 2020, no agents were attending school. After August 24, school-age agents attended school following a hybrid schooling policy [34]. In this policy, the agents were split into two groups. If the day of the simulation was odd, then half of the agents attended school, and if the day was even, then the other half attended school. Attending school corresponds to setting the agents peer group to active.
        - Masks were applied uniformly throughout the population. Masks were worn in the community and peer group settings, not the household. The effectiveness of masks was controlled by two parameters: adherence and efficacy. Adherence refers to the proportions of the agents that wear masks. Efficacy refers to the ability of masks to reduce exposure and infectivity. If an agent wore a mask they were 50% less likely to become exposed to the disease, and 50% less likely to spread the infection [35,36]. Once an agent is assigned a mask, the assignment did not change.
    - Intervention removal occurs exactly halfway through the 6-month vaccine distribution (i.e. 3 months after distribution begins)
        - Probability of voluntary quarantine was set to 0 immediately.
        - The schooling returned to normal. All of the children agents' peer groups were set to active and agents that are not symptomatic attend school. There was not a gradual increase; full school activity resumed immediately.
        - Workplace peer groups returned to normal. All of the agents that could work began attending work. All of the adult agents' peer groups were set to active. There was not a gradual increase; work place activity resumed immediately.
        - Community interaction at the census tract level returned to normal level immediately.
        - Random individuals removed masks as the vaccine was distributed equaling the number of people that had been vaccinated. At the end of the vaccine distribution period, the same number of individuals vaccinated were no longer using face masks.

eFigure 1. Network of Agents Within Households, Schools, Workplaces, and Communities

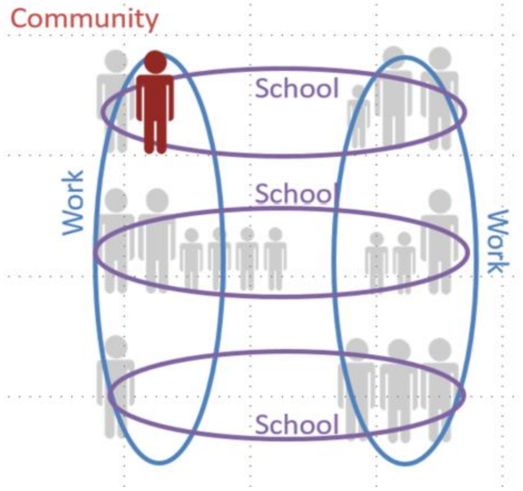

Source: Rosenstrom E, et al. High-Quality Masks Can Reduce Infections and Deaths in the US. *medRxiv*, 2020.

eFigure 2. Time-Varying Workplace Mobility Estimates by Census Tract Urban-Rural Classification and Median Household Income Quartile

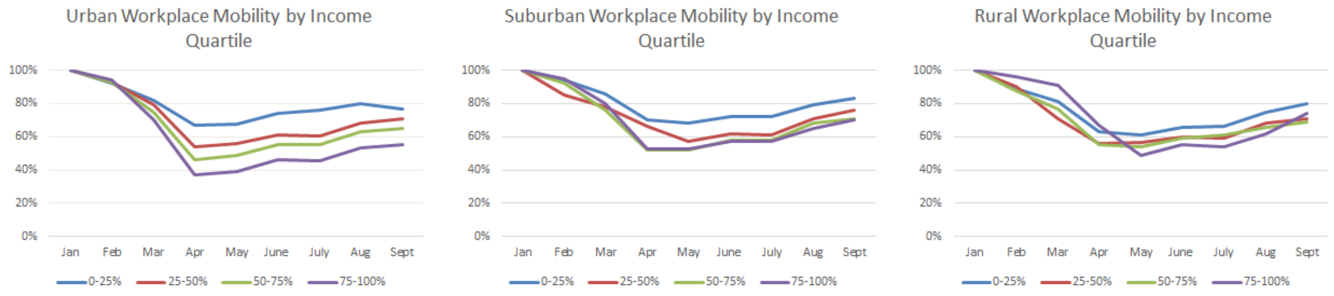

eFigure 3. SEIR Framework for Transition Between Model States

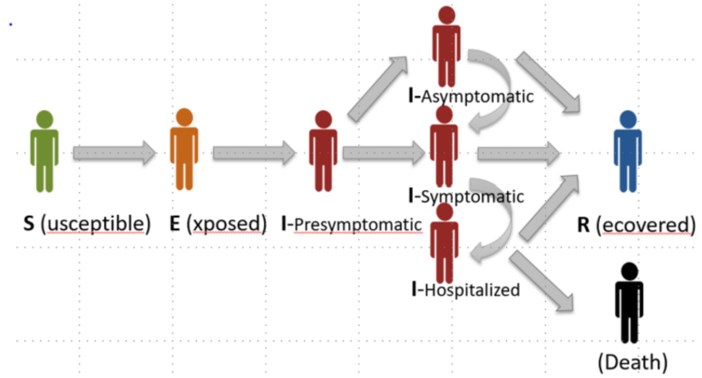

Source: Rosenstrom E, et al. High-Quality Masks Can Reduce Infections and Deaths in the US. *medRxiv*, 2020.

© 2021 Patel MD et al. *JAMA Network Open*.

eTable 1. Key Model Parameters

Parameter	Estimates	References
Exposed (E) Duration	Weibull with mean 4.6 days	[16,17]
Pre-symptomatic (IP) Duration	0.5 days	[16]
Hospitalized (IH) Duration	Exponential with mean 10.4 days	[16,18]
Symptomatic (S) Duration	Exponential with mean 2.9 days	[19]
Symptomatic-Asymptomatic Duration Ratio	1.5	[16]
Probability of Symptomatic (from IP)	0.50-0.82	[20-24]
Probability of Hospitalization without Diabetes	0.005 for age 0-19;<br>0.06 for age 20-64;<br>0.1 for age 65+	[13,14]
Probability of Hospitalization with Diabetes	3 times rates above	
Probability of Death (from IH)	0 for age 0-19;<br>0.0515 for age 20-64;<br>0.3512 for age 65+	[13,14]
R_0	2.4	[25-27]
β transmission rate	1.12	[26]
θ (probability IP to IA)	0.48	[28]
ω (proportion infections by IA)	0.24	[28]
γ (proportion of transmission that occur outside households)	30%	[1,5]
δ (proportion of infections outside households that occur in community)	0.23	[5]; Calibration
FlatImportRate	45	[5]; Calibration
Infection Fatality Rate	0.46%	Results from model transitions with other parameters

eTable 2. Model Validation by Race and RUCA Subgroups

		Cases		Deaths	
		NC Data	Model	NC Data	Model
Race					
	White	61%	61%	65%	63%
	Black	20%	24%	27%	25%
	Other	18%	15%	9%	12%
RUCA					
	Urban	75%	76%	68%	73%
	Suburban	19%	14%	24%	16%
	Rural	7%	10%	8%	11%

© 2021 Patel MD et al. *JAMA Network Open*.

eTable 3. Cumulative Incidence of Infections, Hospitalizations, and Deaths by Vaccination-NPI Scenario Across Racial/Ethnic Groups

Scenarios:	Vaccine		NPIs	Cumulative Infected (%) (SD)			Cumulative Hospitalized (per 100,000) (SD)			Cumulative Mortality (per 100,000) (SD)		
	Efficacy	Coverage		Non-Hispanic White	Non-Hispanic Black	Hispanic	Non-Hispanic White	Non-Hispanic Black	Hispanic	Non-Hispanic White	Non-Hispanic Black	Hispanic
**A1**	90%	75%	Maintained	13.2% (0.2%)	15% (0.3%)	15.1% (0.4%)	484 (12)	555 (19)	475 (25)	64 (3)	73 (6)	60 (8)
**B1**		50%		14% (0.2%)	16.1% (0.3%)	16.1% (0.4%)	517 (12)	606 (20)	503 (26)	68 (3)	79 (6)	64 (8)
**C1**		25%		15.6% (0.2%)	17.6% (0.3%)	17.7% (0.4%)	575 (13)	652 (21)	551 (27)	75 (4)	86 (6)	69 (9)
**D1**	50%	75%		14.3% (0.2%)	16.4% (0.3%)	16.5% (0.4%)	529 (12)	606 (20)	516 (26)	69 (3)	80 (6)	66 (9)
**E1**		50%		15.4% (0.2%)	17.6% (0.3%)	17.5% (0.4%)	569 (12)	655 (21)	544 (27)	75 (4)	85 (6)	70 (9)
**F1**		25%		16.6% (0.2%)	18.6% (0.3%)	18.7% (0.4%)	615 (13)	690 (21)	579 (27)	81 (4)	90 (6)	72 (9)
**G1**	No vaccine			19.1% (0.2%)	21.2% (0.3%)	21.3% (0.4%)	709 (14)	794 (22)	672 (29)	92 (4)	102 (7)	86 (10)
**A0**	90%	75%	Removed	14% (0.2%)	16% (0.3%)	16.1% (0.4%)	513 (12)	583 (20)	504 (37)	68 (3)	78 (6)	65 (9)
**B0**		50%		15.9% (0.2%)	17.8% (0.3%)	17.9% (0.4%)	577 (12)	651 (20)	567 (38)	77 (4)	87 (7)	74 (9)
**C0**		25%		22.6% (0.2%)	24.4% (0.3%)	24.8% (0.4%)	831 (14)	899 (23)	769 (44)	110 (4)	119 (7)	99 (11)
**D0**	50%	75%		17.9% (0.2%)	19.7% (0.3%)	19.9% (0.4%)	653 (13)	715 (21)	616 (39)	86 (4)	95 (7)	81 (9)
**E0**		50%		22.2% (0.2%)	24.1% (0.3%)	24.2% (0.4%)	809 (14)	873 (23)	753 (44)	108 (4)	115 (7)	96 (10)
**F0**		25%		30.2% (0.2%)	31.6% (0.3%)	32.2% (0.4%)	1,112 (15)	1,165 (25)	1,006 (49)	146 (5)	155 (8)	132 (12)
**G0**	No vaccine			46.2% (0.2%)	47.1% (0.3%)	47.6% (0.4%)	1,725 (17)	1,762 (30)	1,515 (60)	220 (6)	226 (10)	194 (15)

Abbreviations: NPI=non-pharmaceutical intervention; SD=standard deviation
Cumulative outcome at the end of the 18-month simulation divided by the total subgroup size

© 2021 Patel MD et al. *JAMA Network Open*.

eTable 4. Cumulative Incidence of Infections, Hospitalizations, and Deaths by Vaccination-NPI Scenario Across Rural-Urban Classification

Scenarios:	Vaccine		NPIs	Cumulative Infected (%) (SD)			Cumulative Hospitalized (per 100,000) (SD)			Cumulative Mortality (per 100,000) (SD)		
	Efficacy	Coverage		Urban	Suburban	Rural	Urban	Suburban	Rural	Urban	Suburban	Rural
**A1**	90%	75%	Maintained	13.7% (0.1%)	13.9% (0.2%)	14.6% (0.2%)	488 (4)	519 (9)	541 (12)	62 (1)	75 (2)	72 (3)
**B1**		50%		14.6% (0.1%)	14.7% (0.2%)	16.1% (0.2%)	522 (4)	549 (9)	604 (12)	66 (1)	78 (2)	85 (3)
**C1**		25%		16.2% (0.1%)	16.3% (0.2%)	17.5% (0.2%)	576 (4)	608 (9)	650 (12)	72 (1)	89 (3)	88 (3)
**D1**	50%	75%		15% (0.1%)	14.8% (0.2%)	16.2% (0.2%)	531 (4)	554 (9)	611 (12)	67 (1)	79 (2)	85 (3)
**E1**		50%		16% (0.1%)	16.3% (0.2%)	17.3% (0.2%)	569 (4)	614 (9)	647 (12)	72 (1)	88 (3)	88 (3)
**F1**		25%		17.1% (0.1%)	17.4% (0.2%)	18.6% (0.2%)	613 (4)	649 (9)	696 (12)	77 (1)	94 (3)	98 (3)
**G1**	No vaccine			19.7% (0.1%)	19.8% (0.2%)	21.5% (0.2%)	704 (4)	747 (10)	811 (13)	88 (1)	107 (3)	112 (4)
**A0**	90%	75%	Removed	14.6% (0.1%)	14.6% (0.2%)	16% (0.2%)	514 (4)	547 (9)	589 (12)	65 (1)	82 (3)	85 (3)
**B0**		50%		16.5% (0.1%)	16.3% (0.2%)	17.6% (0.2%)	580 (4)	605 (9)	646 (12)	74 (1)	87 (3)	91 (3)
**C0**		25%		23.4% (0.1%)	22.1% (0.2%)	24.5% (0.2%)	828 (4)	823 (10)	915 (13)	106 (1)	123 (3)	127 (4)
**D0**	50%	75%		18.5% (0.1%)	18.1% (0.2%)	19.7% (0.2%)	677 (4)	706 (10)	775 (12)	83 (1)	83 (3)	99 (3)
**E0**		50%		22.9% (0.1%)	22.1% (0.2%)	24% (0.2%)	803 (4)	822 (10)	886 (13)	102 (1)	116 (3)	121 (4)
**F0**		25%		30.9% (0.1%)	29.2% (0.2%)	32.3% (0.2%)	1,099 (4)	1,083 (11)	1,203 (14)	138 (1)	157 (3)	171 (4)
**G0**	No vaccine			46.3% (0.1%)	45.4% (0.1%)	50.1% (0.2%)	1,674 (5)	1,711 (12)	1,901 (15)	206 (2)	243 (4)	260 (5)

Abbreviations: NPI=non-pharmaceutical intervention; SD=standard deviation
[1]Cumulative outcome at the end of the 18-month simulation divided by the total subgroup size
[2]Census tract rural-urban commuting area (RUCA) code: 1-2=urban, 3-5=suburban, and 6-10= rural

© 2021 Patel MD et al. *JAMA Network Open*.

eTable 5. Two-Way Sensitivity Analysis of the Risk of SARS-CoV-2 Infection From the Start of Vaccine Distribution by Vaccination-NPI Scenario Across Varying Transmission Rates and Mask Efficacies

Scenarios:	Vaccine		NPIs	Transmission Rate (β)	Mask Efficacy					
	Efficacy	Coverage			70%		50%		30%	
					Risk[1]	RD[2]	Risk	RD	Risk	RD
**A1**	90%	75%	Maintained	1.19	0.040	-0.16	0.063	-0.13	0.071	-0.11
**F1**	50%	25%			0.069	-0.13	0.093	-0.10	0.099	-0.08
**A0**	90%	75%	Removed		0.047	-0.15	0.068	-0.12	0.077	-0.10
**F0 (ref)**	50%	25%			0.198	0.00	0.191	0.00	0.179	0.00
**A1**	90%	75%	Maintained	1.12	0.025	-0.17	0.047	-0.19	0.073	-0.19
**F1**	50%	25%			0.051	-0.14	0.084	-0.15	0.116	-0.14
**A0**	90%	75%	Removed		0.032	-0.16	0.056	-0.18	0.083	-0.18
**F0 (ref)**	50%	25%			0.192	0.00	0.235	0.00	0.260	0.00
**A1**	90%	75%	Maintained	1.06	0.016	-0.13	0.027	-0.13	0.045	-0.24
**F1**	50%	25%			0.034	-0.11	0.057	-0.11	0.086	-0.20
**A0**	90%	75%	Removed		0.020	-0.13	0.035	-0.13	0.056	-0.23
**F0 (ref)**	50%	25%			0.146	0.00	0.22	0.00	0.283	0.00

Abbreviations: NPI=non-pharmaceutical intervention ; RD=risk difference ; ref=referent

[1]From start of vaccine distribution (Day 213) to end of simulation (11.0 months)

[2]Absolute difference in risk compared to the worst-case vaccination scenario and NPIs removed (F0)

© 2021 Patel MD et al. *JAMA Network Open*.

eReferences

1. Ekici A, Keskinocak P, Swann JL. Modeling influenza pandemic and planning food distribution. *Manuf Serv Oper Manag.* 2014;16(1):11-27.
2. Shi P, Keskinocak P, Swann J, Lee B. Modelling seasonality and viral mutation to predict the course of an influenza pandemic. *Epidemiol Infect.* 2010;138(10):1472-1481.
3. Shi P, Keskinocak P, Swann JL, Lee BY. The impact of mass gatherings and holiday traveling on the course of an influenza pandemic: a computational model. *BMC Public Health.* 2010;10(1):778.
4. Keskinocak P, Asplund J, Serban N, Aglar BEO. Evaluating Scenarios for School Reopening under COVID19. *medRxiv.* 2020.
5. Keskinocak P, Oruc BE, Baxter A, Asplund J, Serban N. The impact of social distancing on COVID19 spread: State of Georgia case study. *PLoS One.* 2020;15(10):e0239798.
6. Rosenstrom E, Oruc Aglar B, Ivy JS, Keskinocak P, Mayorga M, Swann JL. High-Quality Masks Can Reduce Infections and Deaths in the US. *medRxiv.* 2020:2020.2009.2027.20199737.
7. US Census Bureau. American Community Survey 5-year Estimates 2017. data.census.gov.
8. US Census Bureau. Census Summary File 1 2010. data.census.gov.
9. America's Health Rankings. Explore Diabetes in North Carolina. https://www.americashealthrankings.org/explore/annual/measure/Diabetes/state/NC.
10. US Census Bureau. Census Transportation Planning Products, 5-year data (2012-2016). data5.ctpp.transportation.org.
11. SafeGraph. Places Data & Foot-Traffic Insights. https://www.safegraph.com/.
12. Google. COVID-19 Community Mobility Reports. https://www.google.com/covid19/mobility/.
13. CDC. COVID-19 Pandemic Planning Scenarios. https://www.cdc.gov/coronavirus/2019-ncov/hcp/planning-scenarios.html. Accessed 12/21/2020.
14. CDC COVID-19 Response Team. Severe Outcomes Among Patients with Coronavirus Disease 2019 (COVID-19)-United States, February 12-March 16, 2020. *MMWR Morb Mortal Wkly Rep.* 2020;69(12):343-346.
15. Hauser A, Counotte MJ, Margossian CC, et al. Estimation of SARS-CoV-2 mortality during the early stages of an epidemic: A modeling study in Hubei, China, and six regions in Europe. *PLOS Medicine.* 2020;17(7):e1003189.
16. Ferguson N, Laydon D, Nedjati Gilani G, et al. Report 9: Impact of non-pharmaceutical interventions (NPIs) to reduce COVID19 mortality and healthcare demand. 2020.
17. Linton NM, Kobayashi T, Yang Y, et al. Incubation period and other epidemiological characteristics of 2019 novel coronavirus infections with right truncation: a statistical analysis of publicly available case data. *Journal of clinical medicine.* 2020;9(2):538.
18. Weitz JS, Beckett SJ, Coenen AR, et al. Modeling shield immunity to reduce COVID-19 epidemic spread. *Nature medicine.* 2020:1-6.
19. Riou J, Hauser A, Counotte MJ, Althaus CL. Adjusted age-specific case fatality ratio during the COVID-19 epidemic in Hubei, China, January and February 2020. *MedRxiv.* 2020;2020.
20. Andrei M. Iceland’s testing suggests 50% of covid-19 cases are asymptomatic. *ZME Science.* 2020.
21. Day M. Covid-19: four fifths of cases are asymptomatic, China figures indicate. *BMJ.* 2020.
22. Mandavilli A. Infected but Feeling Fine: The Unwitting Coronavirus Spreaders. *The New York Times.* 3/31/2020, 2020.
23. Mizumoto K, Kagaya K, Zarebski A, Chowell G. Estimating the asymptomatic proportion of coronavirus disease 2019 (COVID-19) cases on board the Diamond Princess cruise ship, Yokohama, Japan, 2020. *Euro Surveill.* 2020;25(10):2000180.
24. Nishiura H, Kobayashi T, Miyama T, et al. Estimation of the asymptomatic ratio of novel coronavirus infections (COVID-19). *International journal of infectious diseases.* 2020;94:154.
25. *Report of the WHO-China Joint Mission on Coronavirus Disease 2019 (COVID-19).* 16-24 Feb 2020.
26. Li R, Pei S, Chen B, et al. Substantial undocumented infection facilitates the rapid dissemination of novel coronavirus (SARS-CoV-2). *Science.* 2020;368(6490):489-493.
27. Walker PG, Whittaker C, Watson OJ, et al. The impact of COVID-19 and strategies for mitigation and suppression in low-and middle-income countries. *Science.* 2020.
28. Ganyani T, Kremer C, Chen D, et al. Estimating the generation interval for coronavirus disease (COVID-19) based on symptom onset data, March 2020. *Euro Surveill.* 2020;25(17):2000257.
29. The New York Times. Coronavirus in the U.S.: Latest Map and Case Count. https://www.nytimes.com/interactive/2020/us/coronavirus-us-cases.html. Accessed 8/18/2020.

© 2021 Patel MD et al. *JAMA Network Open*.

30. Havers FP, Reed C, Lim T, et al. Seroprevalence of antibodies to SARS-CoV-2 in 10 sites in the United States, March 23-May 12, 2020. *JAMA.* 2020;180(12):1576-1586.
31. NC Department of Health and Human Services. COVID-19 North Carolina Dashboard. https://covid19.ncdhhs.gov/dashboard.
32. The New York Times. Coronavirus in the U.S.: Latest Map and Case Count. https://www.nytimes.com/interactive/2020/us/coronavirus-us-cases.html. Accessed 12/21/2020.
33. Reese H, Iuliano AD, Patel NN, et al. Estimated incidence of COVID-19 illness and hospitalization — United States, February–September, 2020. *Clinical Infectious Diseases.* 2020.
34. NC Department of Health and Human Services. NC.gov: Opening Public Schools in North Carolina. https://www.nc.gov/covid-19/staying-ahead-curve/opening-public-schools-north-carolina. Accessed 12/21/2020.
35. Fischer EP, Fischer MC, Grass D, Henrion I, Warren WS, Westman E. Low-cost measurement of face mask efficacy for filtering expelled droplets during speech. *Sci Adv.* 2020;6(36):eabd3083.
36. Liang M, Gao L, Cheng C, et al. Efficacy of face mask in preventing respiratory virus transmission: A systematic review and meta-analysis. *Travel Med Infect Dis.* 2020;36:101751.

© 2021 Patel MD et al. *JAMA Network Open*.
